# Seroprevalence of Varicella-Zoster Virus and the Need for Herpes Zoster Vaccination Among Adults in Saudi Arabia

**DOI:** 10.7759/cureus.80949

**Published:** 2025-03-21

**Authors:** Omar Alshouimi, Musaad N Al Musaad, Anas Almazyed, Abuobaida Abduraouf, Mostafa Kofi

**Affiliations:** 1 Family and Community Medicine, Prince Sultan Military Medical City, Riyadh, SAU

**Keywords:** herpes zoster, saudi arabia, vaccination, vaccine, varicella, varicella-zoster virus

## Abstract

Introduction: Chickenpox is caused by the varicella-zoster virus (VZV), which can reactivate later in life, leading to herpes zoster (shingles). The varicella vaccine has been integrated into childhood immunization programs in certain regions, contributing to the control and prevention of the disease. However, there remains uncertainty regarding the seroprevalence of varicella among adults, as well as concerns about vaccination rates and public awareness of its importance.

Objective: The objective of this study is to estimate the seroprevalence of varicella among Saudi adults who may require vaccination against varicella and herpes zoster.

Methods: This descriptive cross-sectional study involved 209 Saudi adults aged 18 years and older in Riyadh. Data were collected using a validated questionnaire and serological testing for VZV-IgG antibodies. Statistical analyses were conducted to explore the relationships between seroprevalence, vaccination history, and demographic factors.

Results: The study, comprising primarily young adults aged 18-47 years (83.3%) with a nearly equal gender distribution (52.2% male, 47.8% female), found that 81.1% of participants were serologically immune to VZV. Immunity was higher among males (84.4% vs. 79% in females) and older participants (89.7% in those ≥38 years vs. 78% in those <38 years). A positive history of varicella infection or vaccination increased the likelihood of VZV-IgG positivity by nine times compared to deniers and four times compared to unsure participants. History-based immunity assessment had a sensitivity of 87.1% and specificity of 57.7%.

Conclusion: The study highlights a high prevalence of serological immunity to VZV among Saudi adults. While a positive history strongly predicted immunity, the limited validity of history-based assessments underscores the need for serological confirmation in some cases. Despite widespread immunity and consequently the risk of herpes zoster, the low uptake of herpes zoster vaccination among eligible individuals with a history of shingles points to a gap in preventive healthcare. These findings emphasize the importance of targeted public health efforts to improve vaccination coverage among adults, particularly for herpes zoster.

## Introduction

Varicella-zoster virus (VZV), a member of the *Herpesvirus* family, causes two distinct diseases: varicella (chickenpox) and herpes zoster (shingles) [1]. Varicella primarily affects children, while herpes zoster occurs in older adults or immunocompromised individuals due to VZV reactivation in the dorsal root ganglia [1,2]. Approximately 30% of the population will experience herpes zoster in their lifetime, with incidence increasing with age due to declining VZV-specific immunity [1,2]. In Saudi Arabia, the varicella vaccine was added to the childhood immunization program to prevent primary infection [3]. Before vaccines, varicella was nearly universal in childhood, with complications like bacterial superinfections, pneumonia, encephalitis, and rare deaths [4]. Vaccination has significantly reduced incidence, though outbreaks persist in populations with low coverage [4-6]. It presents as a painful dermatomal rash, with complications like post-herpetic neuralgia (PHN), ophthalmic involvement, and neurological sequelae [2,7]. The global burden of herpes zoster is rising due to aging populations [8]. In Saudi Arabia, data on herpes zoster are limited, but prevalence aligns with global trends, particularly among older adults [9].

The varicella vaccine, introduced in the U.S. in 1995 and in Saudi Arabia in 2008, has high efficacy in preventing varicella and its complications [10,11]. In Saudi Arabia, childhood vaccination coverage is relatively high, but adult vaccination rates remain low due to a lack of awareness and catch-up programs [12]. In Saudi Arabia, the vaccine is free for those over 50, but uptake is low due to limited awareness and misconceptions [12-14]. Screening for VZV immunity involves self-reported histories, serological testing, or both [15]. It is critical for identifying susceptible individuals, tracking vaccination coverage, and monitoring program effectiveness [16]. Serological testing for VZV-specific IgG antibodies is the gold standard for determining immunity. Studies show serological testing can identify asymptomatic or undocumented exposure [17]. For example, a study on Saudi healthcare workers (HCWs) found 83% seropositivity among those with no known history of infection or vaccination [18]. Another study in Riyadh found 11.3% of HCWs susceptible to varicella, highlighting the need for screening [19].

Self-reported histories of varicella infection or vaccination are commonly used but vary in accuracy [20]. A study on Saudi National Guard soldiers found an 88.5% seropositivity rate, even among those without a clear exposure history [21]. Screening for VZV is essential for identifying at-risk individuals, monitoring immunization programs, and managing outbreaks. It enables health authorities to assess community immunity and implement preventive measures, particularly for vulnerable populations [15]. Vaccination is a key preventive measure against many diseases, extending beyond children to adults. While Western studies show high VZV immunity in older adults, limited data exist for Saudi adults. Assessing immunity is essential to determine vaccination needs for primary and secondary infections. Our research is to estimate the seroprevalence of varicella among Saudi adults who may require vaccination against herpes zoster and to evaluate the validity of self-reported history of varicella infection or vaccination among Saudi adults who may require vaccination against herpes zoster.

## Materials and methods

This study follows a descriptive cross-sectional design. The target population included Saudi adults aged 18 and above from Riyadh, with a sample size based on an estimate of 50% seroprevalence and a margin of error of 5% at a 95% confidence level (Z = 1.96), besides accounting for potential non-responses. A convenience sampling method was used to select participants.

Data was collected using a validated questionnaire distributed by electronic form and medical records, including serology testing for VZV-IgG in Prince Sultan Military Medical City (PSMMC), Riyadh. The independent variables included sociodemographic factors (e.g., age, gender) and health status (e.g., history of varicella infection, and vaccination), while the dependent variable was varicella serology with the exclusion of those under 18. Additionally, those who were immunocompromised or immunosuppressed, and that had blood transfused or received immunoglobulin around the time of vaccination were excluded.

The collected data was processed through manual entry, standardization, coding, and classification, followed by data editing, cleaning, and assessing normality. Data was stored securely, with backup and archiving procedures in place. Data analysis was conducted using IBM SPSS Statistics for Windows, Version 22 (Released 2015; IBM Corp., Armonk, New York, United States), where categorical variables were summarized as frequencies and percentages, and differences between proportions were tested using Pearson’s chi-squared test. A significance level of ≤0.05 was set for all tests. The obtained odds ratios (ORs) were later adjusted for possible covariables using logistic regression analysis conducted to ascertain the association between self-reported immunity and VZV serology. Finally, sensitivity, specificity, positive predictive value (PPV), and negative predictive value (NPV) were also calculated to find out the validity and yield of self-reported immunity compared to VZV serology.

## Results

Table 1 summarizes the basic characteristics of the 209 participants. The majority (174 participants, 83.3%) were aged between 18 and 47 years, while 35 participants (16.7%) were over 47 years old. Regarding chickenpox history, 30 participants (14.4%) reported neither having had the infection nor being vaccinated, 112 participants (53.6%) reported a history of either infection (92 participants) or vaccination (20 participants), and 67 participants (32.1%) were uncertain. Additionally, 171 (81.1%) showed positive IgG varicella serology, indicating prior exposure to or vaccination against VZV. A small proportion of participants (11, 5.3%) reported a history of shingles, and only one case of them was vaccinated against herpes zoster after recovery. Approximately eight participants (73%) who reported a history of shingles were over 58 years old. However, while all participants tested positive for VZV-IgG, only 46 (27%) reported a history of varicella infection, while the remaining were unsure whether they had been infected or vaccinated against varicella.

**Table 1:** Basic Characteristics of Saudi Adults Participated in the Study (N=209) VZV: varicella-zoster virus; IgG: immunoglobulin G

Characteristics		N	Percent (%)
Age	18-27 Years	67	32.1
	28-37 Years	74	35.4
	38-47 Years	33	15.8
	>47 Years	35	16.7
Gender	Female	100	47.8
	Male	109	52.2
History of Varicella Infection or Vaccination	Yes	112	53.6
	Not Sure	67	32.1
	No	30	14.4
History of Herpes Zoster Infection or Vaccination	Yes	11	5.3
	No	198	94.7
Result of VZV-IgG Testing	Positive	171	81.8
	Negative	38	18.2

Table 2 reveals notable differences in seroimmunity to VZV based on gender, age, and history of varicella infection or vaccination. Male participants were more likely to be serologically immune to VZV compared to females, with 84.4% of males testing positive for VZV-IgG, compared to 79% of females. Serological immunity also varied by age. Older participants demonstrated higher immunity levels against VZV. Among those aged less than 38 years, 78% tested positive for VZV-IgG, while 89.7% of participants (38 years and over) were serologically immune.

**Table 2:** Basic Characteristics by Seroimmunity to Varicella-Zoster Virus (VZV) Among Saudi Adults (N=209) IgG: immunoglobulin G; OR: odds ratio; CI: confidence interval * P-value was considered significant if ≤ 0.05.

Characteristics		VZV-IgG		OR (95% CI)	P-value*
		Positive	Negative		
Gender	Male	92	17	1.44 (0.71, 2.92)	0.312
	Female	79	21		
Age	18-27 Years	49	18	0.45 (0.15,1.35)	0.071
	28-37 Years	61	13	0.78 (0.26, 2.40)	
	38-47 Years	31	2	2.58 (0.47, 14.35)	
	>47 Years	30	5	-	
History of Varicella Infection or Vaccination	Yes	101	11	9.18 (3.56, 23.70)	0.004
	Not Sure	55	12	4.583 (1.77, 11.85)	
	No	15	15	-	
History of Herpes Zoster Infection	Yes	11	0	-	-
	No	160	38		

History of varicella infection or vaccination was strongly associated with seroimmunity. Participants who reported a positive history of varicella infection or vaccination were significantly nine times more likely to be serologically immune to VZV compared to those who denied any history. Even among those who were unsure or could not recall their history, the likelihood of seroimmunity was significantly four times higher compared to deniers (p < 0.05). Specifically, 90.2% of participants with a positive history of varicella infection or vaccination were serologically immune, compared to 82.1% of the unsure group and 50% of the deniers.

Regarding herpes zoster, 80.8% of participants who had not been infected were at risk of developing it. The remaining 19.2% were susceptible to varicella, which could lead to herpes zoster if they contracted chickenpox or were vaccinated. Interestingly, all participants who had experienced shingles were found to be serologically immune to VZV.

Table 3 presents a logistic regression that was conducted to evaluate the association between self-reported history of varicella infection or vaccination and serological immunity to VZV. The model was adjusted for potential co-variables, including age and gender. The analysis revealed that self-reported history of varicella infection or vaccination was independently associated with serological immunity. Participants who confidently reported a history of varicella infection or vaccination were significantly more likely to test positive for VZV-IgG compared to those who denied such a history. Interestingly, even participants who could not recall or were unsure of their history showed a higher likelihood of testing positive for VZV-IgG, suggesting that uncertainty does not preclude immunity.

**Table 3:** Predictors of Serological Immunity of Varicella-Zoster Virus (VZV) Among Saudi Adults (N=209) OR: odds ratio; CI: confidence interval

Characteristics		Adjusted OR (95% CI)	P-value
Age	18-27 Years	0.31 (0.09, 1.06)	0.061
	28-37 Years	0.50 (0.15, 1.73)	0.276
	38-47 Years	3.40 (0.55, 21.06)	0.188
Gender	Male	1.55 (0.69, 3.51)	0.293
Self-Reported History of VZV Infection or Vaccination	Yes	14.29 (4.99, 40.88)	<0.001
	Not Sure	4.71 (1.66, 13.37)	0.004

Table 4 presents the validity and yield of immunity assessment based on history, using the prevalence of varicella in this study (81%). The estimation was conducted in three steps: first, by excluding individuals who reported being unsure of their immunity status (Value 1); second, by classifying those unsure as non-immune by history (Value 2); and third, by classifying them as immune by history (Value 3).

**Table 4:** Validity and Yield of Screening for Immunity of Varicella Based on History Among Saudi Adults CI: confidence interval; LH+: positive likelihood ratio; LH-: negative likelihood ratio; PPV: positive predictive value; NPV: negative predictive value

Statistic	Value 1 (95% CI) (N=142)	Value 2 (95% CI) (N=209)	Value 3 (95% CI) (N=209)
Sensitivity	87.1% (79.6%, 92.6%)	59.1% (51.3%, 66.5%)	91.2% (85.9%, 95.0%)
Specificity	57.7% (36.9%, 76.7%)	71.1% (54.1%, 84.6%)	39.5% (24.0%, 56.6%)
LH+	2.1 (1.3, 3.2)	2.0 (1.2, 3.4)	1.5 (1.2, 2.0)
LH-	0.2 (0.1, 0.4)	0.6 (0.4, 0.8)	0.2 (0.1, 0.4)
Prevalence	81.7% (74.3%, 87.7%)	81.8% (75.9%, 86.8%)	81.8% (75.9%, 86.8%)
PPV	90.2% (85.4%, 93.5%)	90.2% (84.6%, 93.9%)	87.2% (83.9%, 89.8%)
NPV	50.0% (36.0%, 64.0%)	27.8% (22.7%, 33.6%)	50.0% (34.9%, 65.1%)
Accuracy	81.7% (74.3%, 87.7%)	61.2% (54.3%, 67.9%)	81.8% (75.9%, 86.8%)

When individuals who were unsure were excluded, the sensitivity of immunity by history, using VZV-IgG results as the standard for confirmatory diagnostic tests, was 87.1%, and the specificity was 57.7%. This indicates that the probability of correctly identifying an immune individual based on history was 0.87, while the probability of correctly identifying a non-immune individual was 0.58. These sensitivity and specificity values yielded a positive likelihood ratio (LH+) of 2.1 and a negative likelihood ratio (LH-) of 0.2. Additionally, the PPV and NPV were 90.2% and 50%, respectively. This means that the probability of VZV-IgG positivity when a person reported a positive history was 0.9, while the probability of VZV-IgG negativity when a person reported a negative history was 0.5. Overall, these findings suggest that the probability of correctly classifying a patient’s immunity status based on history alone is approximately 82%.

When unsure participants were classified as immune, the sensitivity increased, but the specificity decreased. Additionally, the PPV decreased, while the NPV remained unchanged. Conversely, when unsure participants were classified as non-immune, the sensitivity decreased, and the specificity increased. In this scenario, the PPV remained unchanged, whereas the NPV decreased.

## Discussion

Varicella infection typically provides lifelong immunity, and reinfection is rare. However, latent VZV reactivation can lead to severe complications, including pneumonia, encephalitis, and postherpetic neuralgia [22]. Transmission occurs through direct contact with vesicular fluid, aerosolized droplets, or rash lesions of infected individuals. The introduction of varicella vaccination has significantly reduced the incidence and complications associated with primary infection [4].

Herpes zoster primarily affects older adults (>55 years) and immunocompromised individuals, with an annual global incidence ranging from 1.2 to 3.4 cases per 1,000 people among healthy individuals [23]. Although global data on herpes zoster prevalence are available, data specific to Saudi Arabia remain scarce. Notably, the incidence of herpes zoster is increasing worldwide, especially in aging populations. Shingles and its complications are largely preventable through the herpes zoster vaccine, a safe and effective intervention recommended for individuals aged 50 years and older. The vaccine, provided free of charge to eligible individuals in Saudi Arabia, is most effective when administered in a two-dose schedule [24].

Despite the availability of effective vaccines, herpes zoster vaccination rates remain low in many countries, including Saudi Arabia [9]. Barriers to vaccine uptake include sociodemographic factors such as age, gender, education, income, and access to primary care services. Cultural and religious beliefs may also influence vaccine acceptance. These barriers underscore the need for targeted public health strategies to increase vaccination coverage and awareness of the benefits of preventing herpes zoster and its complications.

In this study, we aimed to estimate the seroprevalence of varicella among immunocompetent Saudi adults, providing insights into community immunity levels and informing vaccination policy. Our findings contribute to understanding the burden of VZV-related diseases in Saudi Arabia and highlight the importance of improving vaccination efforts to mitigate the risks associated with both varicella and herpes zoster.

Our study, which assessed the varicella vaccination status of 209 Saudi adults, revealed important findings on VZV exposure and immunity within the population. Among the participants, 14.4% reported neither having had the infection nor vaccination, 53.6% reported a history of infection or vaccination, and 32.1% were uncertain. Serological testing showed that 81.1% of participants had positive IgG results, indicating prior exposure to or vaccination against VZV. Notably, only 5.3% of participants reported a history of shingles, and just one of these had received the herpes zoster vaccine post-recovery.

Our results align with previous studies in Saudi Arabia and the region, which have consistently shown varying rates of seroprevalence and vaccination. For instance, AlMuammar et al. reported that while 57.2% of participants were aware of the shingles vaccine, only 7.7% had received it, and 53.2% expressed willingness to be vaccinated [12]. This highlights a gap between awareness and actual vaccination uptake, mirroring our findings of low varicella vaccination rates despite high seroprevalence. Similarly, a recent study noted that only 4.5% of Saudi adults had received the varicella vaccine, a figure too low to achieve significant population-level immunity through vaccination alone [9].

Historical data further support these trends. A 1999 study among HCWs in Saudi Arabia found that 64% had a history of chickenpox, 25% were unaware of their status, and 11% reported no history of exposure [18]. Interestingly, 83% of HCWs with negative or unknown exposure history were serologically immune to VZV, indicating widespread prior exposure. Another seroprevalence study in Riyadh showed that 11.3% of HCWs were susceptible to varicella, raising concerns about potential outbreaks in healthcare settings [25].

Age-related increases in seroprevalence have also been consistently observed. A study by Hossain reported an overall seroprevalence of 68%, with rates increasing progressively with age, reaching 90% in adults [26]. This age-related trend was echoed in a study of Saudi National Guard soldiers, which demonstrated a seropositivity rate of 88.5% [21]. Similarly, a study involving Saudi pregnant women found that 74.4% had VZV-IgG antibodies, further supporting the notion of widespread exposure across different demographic groups [27].

Regional studies reinforce these findings. In the UAE, a study reported that 19.4% of children and healthy adults were susceptible to VZV, with an overall adult seroprevalence rate of 81.3% [28]. Seroprevalence increased with age, from 45.8% in children under 10 years to 94.7% in adults aged 31-40 years. These results are consistent with our findings, which also demonstrated significant relationships between age, history of infection, and IgG serology results.

Our findings emphasize the high seroprevalence of VZV in Saudi Arabia, driven largely by natural infection rather than vaccination. Despite the high seroprevalence, the low rates of vaccination against both varicella and herpes zoster remain concerning. Even among participants who were uncertain about their history of infection or vaccination, a substantial proportion tested positive for VZV-IgG, suggesting the need for more comprehensive serological screening and vaccination programs. These efforts are critical to bridging the gap between awareness and uptake, particularly for high-risk groups such as older adults and HCWs.

## Conclusions

The findings emphasize the critical role of serological testing to confirm immunity, especially in cases where self-reported history is uncertain or negative. Despite the high prevalence of natural immunity, the low uptake of herpes zoster vaccination among eligible individuals highlights a substantial gap in preventive healthcare. The low vaccination coverage or high proportion of uncertainty about vaccination status among eligible individuals highlights the need for improved public education and outreach to raise awareness of the benefits and accessibility of vaccination. Given the high prevalence of herpes zoster, it is imperative for the health authorities to prioritize vaccination and implement strategies to enhance immunization coverage. Such measures are critical to reducing the morbidity and complications associated with VZV infection and reactivation in the population.
